# ViTCNN: a robust hybrid CNN–Vision Transformer based deep learning framework for multi-disease diagnosis in women’s healthcare

**DOI:** 10.3389/fonc.2026.1746864

**Published:** 2026-03-25

**Authors:** Sonam Juneja, Bhoopesh Singh Bhati, Ghanshyam G. Tejani, Seyed Jalaleddin Mousavirad

**Affiliations:** 1 Department of Computer Science and Engineering, Indian Institute of Information Technology, Sonepat, India; 2 Department of Industrial Engineering and Management, Yuan Ze University, Taoyuan, Taiwan; 3 Applied Science Research Center, Applied Science Private University, Amman, Jordan; 4 Department of Computer and Electrical Engineering, Mid Sweden University, Sundsvall, Sweden

**Keywords:** breast cancer, cervical cancer, diagnostic image classification, EfficientNetB0, hybrid deep learning, multi-disease identification, polycystic ovary syndrome (PCOS), Vision Transformer (ViT)

## Abstract

Accurate and efficient detection of multiple diseases from diagnostic images remains a major challenge in today’s world, especially in women’s health conditions such as breast cancer, cervical cancer, and Polycystic Ovary Syndrome (PCOS). Each of these diseases presents its own unique imaging characteristics and visual patterns, making detection of these diseases all together through a single model is highly challenging. In this respect, in order to overcome this, we have proposed a hybrid deep learning framework that combines EfficientNetB0 and Vision Transformer for multiple multi-disease detection. This shared backbone and multi-head architecture of the proposed framework integrate the strong spatial feature extraction ability of EfficientNetB0 with the contextual reasoning ability of the Vision Transformer, ensuring that the model is able to capture both local and global features of diseases. Our framework was trained on a different dataset containing several thousand of annotated diagnostic images using a two-stage learning strategy: 70 epochs of initial training followed by 30 epochs of fine-tuning. Experimental results show very impressive diagnostic performance, where our approach has achieved accuracies of 97.64% for breast cancer, 94.28% for cervical cancer, and 98.10% for PCOS. These numbers are improved to 98.82%, 95.96%, and 98.96%, respectively, after a fine-tuning stage. Future work on this study will focus on dataset expansion and clinical validation for real-world diagnostic deployment.

## 1 Introduction

The combination of artificial intelligence (AI) in diagnostic imaging is showing how the complex diseases are detected, managed, and treated. Among women’s health conditions, diseases such as breast cancer, cervical cancer, and polycystic ovary syndrome (PCOS) share a crucial diagnostic challenge—each relies on visual imaging and manual analysis by clinicians. Traditional diagnostic procedures, including ultrasound, cytology, and histopathology, are highly dependent, often leading to inconsistent outcomes and delayed treatment. This has shown the transition toward the multi-disease prediction, which is capable of detecting all the three diseases in a single model.

To the date, existing deep learning (DL) models in women’s health have largely dependent on single-disease prediction, leaving a large research gap in the multi-disease diagnostic systems. To the best of our knowledge, this study is the first to introduce a deep learning framework capable of simultaneously screening for and classifying multiple diseases specifically diseases such as breast cancer, cervical cancer, and PCOS—from diagnostic images. This work presents a general approach for women’s health diagnostic conditions by integrating the multiple disease-specific datasets into a single, adaptive prediction pipeline.

In the context of women’s health, PCOS is a prevalent chronic endocrine disorder affecting women of reproductive age and remains an important target for computer-aided diagnosis and clinical decision support, affecting nearly 13% of reproductive-aged women. Divekar et al. introduced an AI-based classification pipeline under the AUTOPCOS Challenge, it uses transfer learning with InceptionV3 and explainability tools such as LIME and saliency maps, achieving 90.52% accuracy on ultrasound images (1). Reka et al. later extended this work through the QEI-SAM framework, which enhanced imaging using ESRGANs, employed cyst segmentation via the SAM model, and applied CNN-based classifiers such as VGG19, ResNet-101, and InceptionV3—achieving accuracies exceeding 99% (2). While these studies show the power of deep learning, their models remain disease specific, limiting their adaptability across multiple diagnostic conditions.

Meanwhile, advances in neural architectures have gradually improved the model strength and accuracy. ResNet, introduced by He et al., proved vanishing-gradient issues through residual connections, enabling the deeper networks which is capable of learning complex multiple features (3). EfficientNet, developed by Tan and Le, optimized the convolutional networks through the compound scaling method, by balancing the depth, width, and input resolution for maximum performance of an model (4). Similarly, the Vision Transformer (ViT) by Dosovitskiy et al. have clearly shown that attention-based architectures should outperform the convolutional models by capturing long-range dependencies and global contextual information (5). Each of these architectures brings an unique advantage: CNNs specialize in local texture and edge detection, while transformers show the betterment in understanding the structural and contextual relationships among all other models.

In clinical imaging applications, the CNNs and transformers have both achieved good results. For example, hybrid CNN models have shown the good performance in skin lesion classification (6). Likewise, the transformer-augmented CNNs have improved colorectal lesion analysis (7). However, the majority of such studies remain contributed to single-disease analysis, lacking the generalization necessary for multi-disease diagnostic applications.

To bridge this gap, the present work introduces a multi-disease prediction framework designed to handle multiple imaging datasets related to women’s health. The proposed system integrates the strengths of convolutional and transformer-based architectures to extract both localized and global features, ensuring consistent diagnostic accuracy across different disease categories. By training the model on datasets representing breast cancer, cervical cancer, and PCOS, the framework shows its ability to adapt across domains and maintain understandability; it is a crucial factor in diagnostic AI adoption.

**Figure 1:**
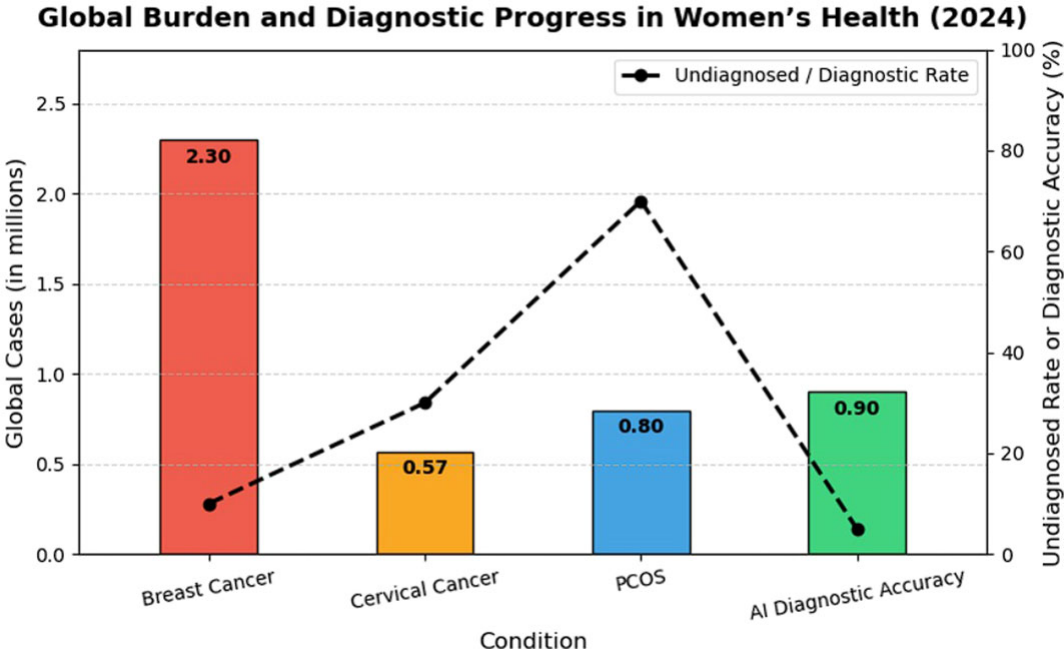
Global burden and diagnostic progress in women’s health conditions (2024).

The prevalence of three serious diseases affecting women worldwide, including PCOS, breast cancer, and cervical cancer, as well as the advancements in AI-based solutions, are displayed in this **Figure 1**. About 2.3 million women are affected by breast cancer each year, compared to 0.57 million for cervical cancer and 0.8 million for PCOS. However, there is large diagnostic gaps revealed by the fact that up to 70% of PCOS cases and 30% of cervical cancer cases go unnoticed. With an accuracy that is still not very high, AI systems have a lot of room to grow. In order to provide early, accurate, and unified women’s health diagnostics, a multi-disease detection framework integrating AI is therefore needed in today’s world.

**Figure 2:**
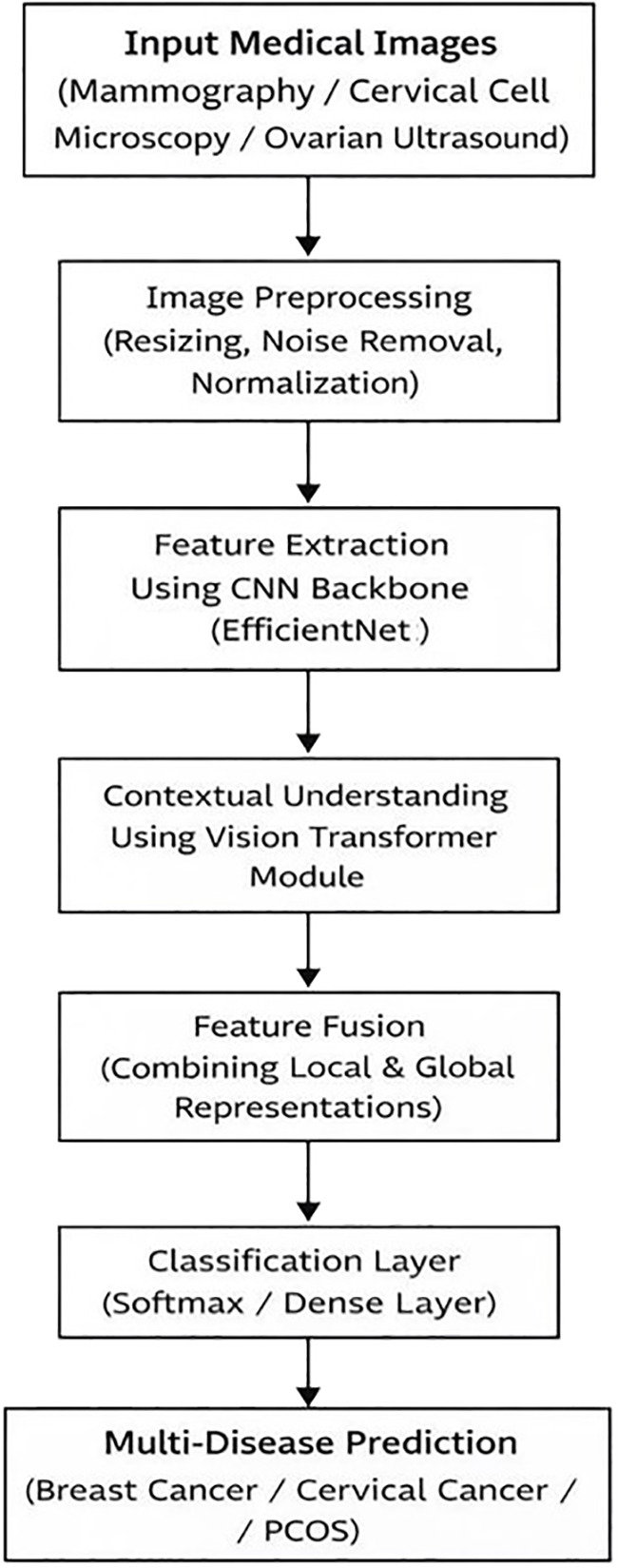
Flowchart of the proposed multi-disease prediction framework for women’s health diagnostics.

**Figure 2** shows the proposed multi-disease prediction framework follows a procedural pipeline for automated screening and diagnosis of women’s health conditions such as breast cancer, cervical cancer, and PCOS. Diagnostic images from different types of datasets are first collected and stored. The preprocessing stage includes resizing, denoising, and normalization to ensure consistency across all three datasets. The processed images are then transferred into a CNN backbone (EfficientNet) to extract detailed local and spatial features. These are passed to a Vision Transformer (ViT) module that captures long-range contextual relationships within the image. The outputs from both networks are forced to form a detailed feature representation combining local precision with global understanding. A dense classification layer with softmax activation predicts the specific disease type among breast cancer, cervical cancer, and PCOS. Finally, the modules such as Grad-CAM and LIME shows the important image regions showing predictions, ensuring transparency and trust in AI-driven medication. For women’s healthcare, this model provides a quality and accurate multi-disease screening and classification.

### 1.1 Main contributions

The main contributions of this work are as follows: (i) we propose a unified multi-disease hybrid CNN-Transformer architecture capable of simultaneously detecting breast cancer, cervical cancer, and PCOS within a single framework; (ii) we introduce a shared feature extraction backbone with task-specific classification heads that enables knowledge transfer across heterogeneous diagnostic imaging modalities; (iii) we present a detailed and reproducible architecture design including tokenization strategy, fusion mechanism, and multi-task optimization formulation; and (iv) we demonstrate consistent performance improvements over conventional CNN and ViT baselines across all three disease tasks. These contributions distinguish the proposed approach from existing single-disease and single-modality systems.

## 2 Literature survey

The sudden growth of deep learning in diagnostic imaging has increased disease prediction and its automated diagnostic and screening techniques, providing a new dimension comparing with the traditional manual analysis. Convolutional Neural Networks (CNNs) and transformer-based models have shown their excellent capabilities in feature extraction, pattern recognition, and semantic segmentation across various diagnostic datasets. However the most existing systems are designed for single-disease prediction. This challenge has motivated the research toward the hybrid architectures that integrate convolutional feature extraction with transformer-based global reasoning, enabling deeper and broader disease understanding. Recent work has shown that combining CNN’s spatial feature learning with the global self-attention capability of the Vision Transformer (ViT) allows for a more detailed analysis of diagnostic images (6). The multi-disease frameworks have emerged as the promising solution for cross-condition prediction, particularly in applications involving cancer detection, skin lesion analysis, and reproductive health diagnostics such as Polycystic Ovary Syndrome (PCOS).

Among women’s health conditions, PCOS affects nearly 10% of women of reproductive age (8) and remains a major focus for computeraided treatment. Reka et al. (2) proposed a combined approach, QEI-SAM (Quality Enhanced Image–Segment Anything Model), which analyzed ultrasound images using ESRGAN and performed cyst segmentation via SAM. This method has achieved a Dice coefficient of 0.9501 and an IoU score of 0.9050, showing that the image enhancement and segmentation prior to classification gradually improve diagnostic performance. Similarly, studies using hybrid CNN architectures such as ResNet-50, ResNet-101, and InceptionV3 have shown their greater performance in detecting cystic ovarian patterns, achieving an accuracy up to 99.3% (2). Moral et al. further developed the CystNet, an automated PCOS classification framework combining InceptionV3 with convolutional autoencoders for follicle segmentation, achieving 97.75% accuracy (9). These frameworks shows how the advanced feature extraction and segmentation increase the precision in disease-specific imaging, forming the first step toward broader multi-disease systems.

Parallel innovation in oncological imaging has shown the strength of CNN–Transformer hybrids for multi-disease diagnostic adoption. Nie et al. (6) introduced a CNN–Transformer model employing focal loss for dermoscopic skin lesion classification, combining ResNet-50 feature extraction with a ViT encoder to achieve great results on the ISIC 2018 dataset while addressing the class imbalance in it. Similarly, Sathyanarayana et al. (7) developed ColoViT, a synergistic EfficientNet–ViT model for colorectal lesion diagnosis that captured both local and global features accurately. EfficientUNetViT (8) further extended this idea by combining pre-trained ViT modules with EfficientNet convolutional blocks for breast tumor segmentation, achieving faster convergence and higher accuracy. These approaches validate the scalability of hybrid models and demonstrate their applicability in multi-disease prediction scenarios. Recent hybrid CNN-Transformer studies further confirms that effectiveness of combining the convolutional feature extraction with an attention based global reasoning for diagnostic image analysis (10–12). Hayat and Aramvith (13) introduced a Superpixel-Guided Graph-Attention Boundary GAN that combines graph attention, residual attention, and adaptive feature refinement to achieve strong segmentation under weak supervision, showing the importance of boundary-aware feature modeling and global context integration. In addition, detail investigations on transformers in diagnostic imaging (14) report that self-attention mechanisms consistently improve representation learning and generalization across multiple modalities, supporting the adoption of transformer encoders within hybrid CNNTransformer pipelines. These findings strongly motivate the architectural design of the proposed EfficientNet–Vision Transformer framework.

Foundational works on CNN and transformer architectures have shown their way for these developments. EfficientNet, introduced by Tan and Le, utilized compound scaling to balance network depth, width, and resolution, achieving an optimal accuracy-efficiency trade-off (4). ResNet, proposed by He et al., introduced residual connections to develop the degradation problem in deep learning-based neural networks, ensuring a stable gradient propagation and deeper hierarchical learning (3). Meanwhile, the Vision Transformer (ViT) by Dosovitskiy et al. extended the self-attention mechanism to vision tasks, treating images as the sequences of patches to capture long-range dependencies (5). This architectural research has shaped the modern frameworks for multi-disease diagnostic image analysis, combining CNN efficiency with transformer contextual awareness to improve diagnostic performance and generalization.

Comparative studies further introduced the importance of hybrid and generalizable architectures. Herman et al. (15) evaluated VGG-16, DenseNet121, ResNet50V2, and EfficientNetB0 cross multiple classification tasks in a non-medical domain and found that EfficientNetB0 achieved the highest accuracy (98%), while ResNet50V2 showed better cross-domain generalization. Similar trends have been reported in studies involving bladder lesion detection and broader multi-disease classification, where EfficientNet–ViT hybrids have consistently shown diagnostic improvements with reduced computational cost (16). These findings show that deep learning’s next frontier lies not merely in disease-specific specialization but in multi-disease frameworks capable of adapting to diverse conditions and datasets. Karimi et al. (17) presented a comprehensive survey on feature selection methods in large-scale diagnostic databases and demonstrated that optimal feature representation plays a crucial role in improving classification accuracy and model interpretability for diagnostic image analysis. Their findings highlight the importance of designing architectures that can learn discriminative and compact features for reliable disease prediction.

Mughal et al. (18) proposed a novel breast tumor classification scheme aimed at reducing mortality among women by improving automated diagnostic accuracy. In a related study, Mughal et al. (19) introduced a pectoral muscle removal technique based on topographic mapping and shape-shifting silhouettes, showing that effective preprocessing and structural isolation significantly enhance mammographic image quality and downstream classification performance.

Saba et al. (20) developed a cloud-based decision support system for malignant cell detection using breast cytology images, demonstrating the potential of intelligent platforms for assisting clinicians in automated diagnosis. Nasir et al. (21) further extended this direction by introducing a CNNenabled cloud and blockchain-based access control framework for secure skin cancer detection. Yousaf et al. (22) reviewed mobile-health applications for efficient healthcare delivery and clinical decision support, highlighting the growing role of intelligent systems in modern healthcare. Although these studies focus on individual applications, they motivate the development of unified multi-disease learning frameworks such as the one proposed in this work.

**Table 1** shows a comparative summary of key studies on multi-disease detection using deep learning models. It shows how the researchers have applied the hybrid CNN–Transformer architectures and enhancement models to improve the diagnostic accuracy. Methods like QEI-SAM and CystNet show strong results in PCOS detection, while ResNet–ViT and EfficientNet–ViT perform excellently for skin and colorectal lesion analysis. Across studies, accuracies consistently exceed 95%, proving the use case of hybrid feature extraction and attention mechanisms. Overall, the table shows the growing trend toward generalized, multi-disease frameworks that outperform traditional single-disease systems.

**Table 1:** Summary of recent related works on multi-disease detection using deep learning models.

Author/year	Model/Method and application	Accuracy (%)
Reka et al.(2025) (2)	QEI-SAM (ESRGAN + Segment Anything Model) for PCOS cyst segmentation and classification using ultrasound images	95.01 (Dice)
Moral et al.(2024) (9)	CystNet (InceptionV3 + Autoencoder) for automated PCOS detection from ovarian ultrasound images	97.75
Nie et al. (2023) (6)	ResNet-50 + Vision Transformer (CNN–Transformer Hybrid) for skin lesion classification (ISIC 2018)	96.2
Sathyanarayana et al. (2025) (7)	EfficientNet + Vision Transformer (ColoViT) for colorectal lesion classification using colonoscopy images	98.1
Herman et al. (2024) (15)	Comparative analysis of VGG16, DenseNet121, ResNet50V2, and EfficientNetB0 for cross-domain diagnostic image classification	98.0

## 3 Proposed methodology

This study presents an integrated deep learning framework combining the EfficientNetB0 network as a basic architecture with the Vision Transformer (ViT) for multi-disease prediction in women’s health regarding breast cancer, cervical cancer, and PCOS. The proposed methodology introduces a single feature extraction backbone with task-specific classification heads to enable simultaneous and efficient disease detection in a single model. It captures the long dependency pattern from the images and performs disease classification within a unified model rather than using each model for each case.

### 3.1 Datasets

The proposed model was trained, and performance was noticed on the three open-source datasets representing major women’s health conditions—breast cancer, cervical cancer, and polycystic ovary syndrome (PCOS).

• CBIS-DDSM (Breast Cancer): It contains the mammography scan images categorized as benign and malignant, serving as a stable performance for breast cancer classification.
• Cervical Cancer Screening Dataset: It contains the cervical cell images, which is classified into three lesion types, showing evaluation of multi-class diagnostic performance.
• PCOS Ultrasound Dataset: It consists of greyscale ovarian ultrasound images which are described as *PCOS* or *Non-PCOS*

The total dataset contains 25,767 images in total. A fix split strategy was used for the model development:

• Training Set: 18,041 images are used for are used for learning the shared and task-specific model parameters.
• Validation Set: 15% of the images ( ≈3,863) are used for fine tuning and monitoring the training progress during model building process.
• Test Set: remainder images are employed to provide an objective final assessment while preserving the initial class distribution for every disease category.

The combined dataset contains approximately 25,767 images pooled from three public datasets. A global 70/15/15 split strategy was applied, resulting in a combined test set of approximately 3,863 images. For reporting disease-wise performance, task-specific test sets were constructed from the combined test pool while preserving the original class distributions, resulting in N = 254 breast cancer images, N = 297 cervical cancer images, and N = 578 PCOS images. **Table 2** shows the dataset-wise test set sizes and the corresponding evaluation subsets used for reporting disease-specific performance.

**Table 2:** Dataset-wise test set size and evaluation subsets used for reporting performance.

Dataset	Test set size	Evaluation subset used
CBIS-DDSM (Breast Cancer)	∼1,530	254
Cervical Cancer Screening	∼1,755	297
PCOS Ultrasound Dataset	∼578	578
Combined Pool	3,863	1129

### 3.2 Architectural overview of the proposed hybrid system

**Figure 3:**
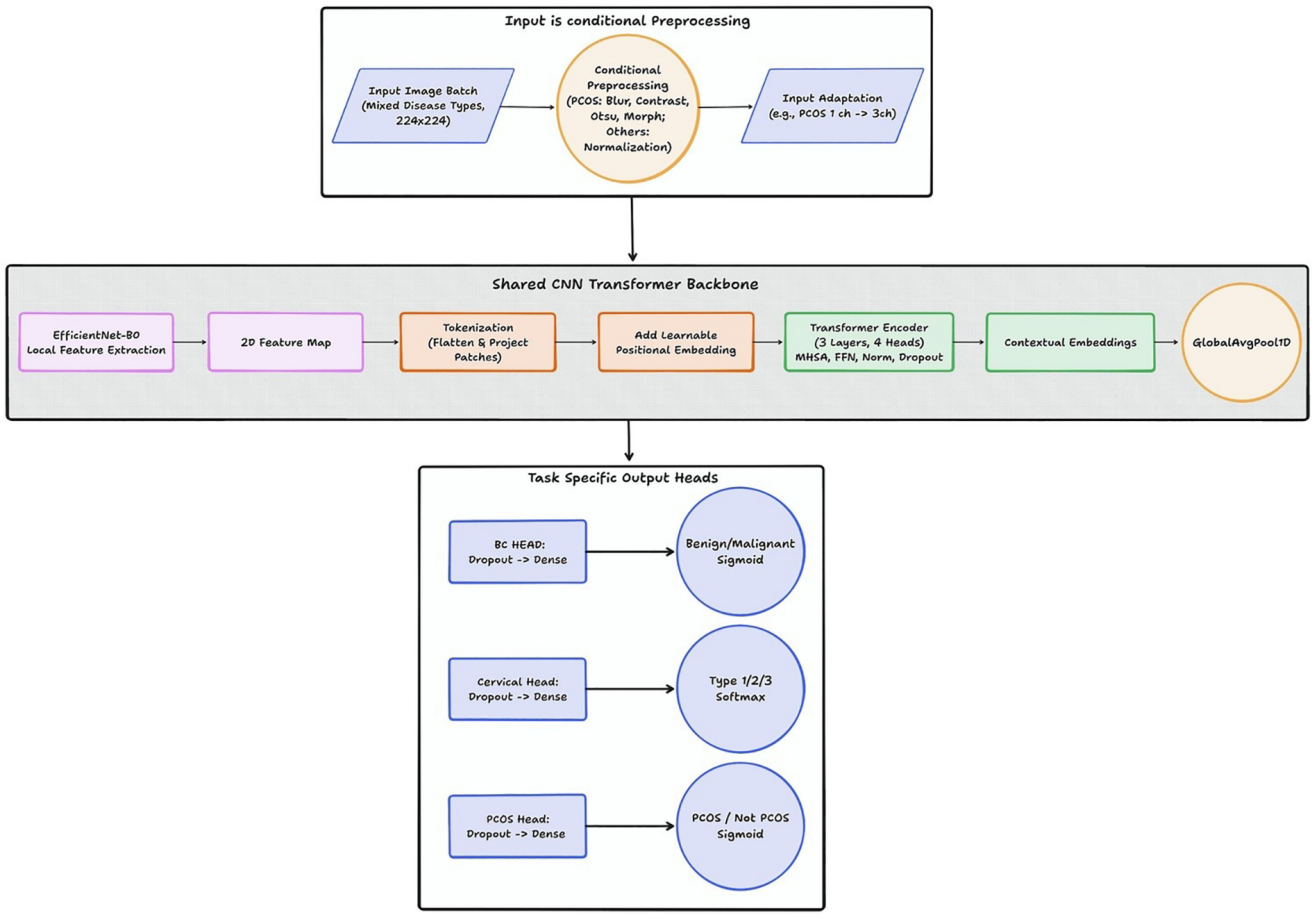
The suggested hybrid CNN-Transformer model’s architecture.

The model introduces a multi deep learning framework designed to perform multi-disease detection across three major women’s health conditions—breast Cancer, cervical cancer, and polycystic ovary syndrome (PCOS)—using diagnostic images. The architecture is built to overcome a fundamental challenge in clinical imaging research: although these diseases originate from different organs and imaging modalities, integrating them into a single multi-task learning system allows the model to share representational knowledge while still maintaining disease-specific decision pathways as shown in **Figure 3**, is divided into three functional modules: conditional preprocessing, a shared CNN–Transformer backbone, and task-specific output heads.

#### 3.2.1 Input and conditional preprocessing

The pipeline begins with a batch of diagnostic images derived from the CBIS-DDSM Breast Cancer Dataset, the Cervical Cancer Screening Dataset, and the PCOS Ultrasound Dataset.

The system first identifies the dataset type and applies conditional preprocessing:

• Mammography scans from the CBIS-DDSM Dataset undergo depth normalization, contrast enhancement, and noise suppression to preserve gradients and micro-calcifications.
• Images from the Cervical Cancer Screening Dataset benefit from color normalization, brightness correction and occasionally vessel enhancement to highlight epithelial changes
• The pipeline applies adaptive contrast enhancement and Gaussian smoothing to display follicular boundaries and ovarian textures present in the PCOS Dataset.

#### 3.2.2 Shared CNN–Transformer backbone

The core of the proposed architecture is a shared CNN–Transformer backbone that integrates the strengths of both Convolutional Neural Networks (CNNs) and Transformers to learn rich representations from the CBIS-DDSM Breast Cancer Dataset, the Cervical Cancer Screening Dataset, and the PCOS Ultrasound Dataset. The pipeline begins with EfficientNet-B0, which extracts detailed 2D spatial feature maps capturing key localized patterns such as micro-calcifications in mammograms, lesion borders in cervical images, and follicle or cystic structures in ultrasound scans. These feature maps are then divided into patches and tokenized so they can be processed as sequential inputs by the transformer module. To preserve the spatial relationships within the image, each token is introduced with learnable positional embeddings, enabling the transformer to understand the original layout of the diagnostic structures. The token sequence is now passed through three Transformer Encoder layers with four attention heads, allowing the model to capture complex long-range dependencies such as architectural Data degradation in breast tissue, which is distributed in a lesion characteristics in cervical imagery, and widespread follicular arrangements in ovarian scans.

#### 3.2.3 Feature tokenization and CNN-Transformer fusion

Let $F\in {\mathbb{R}}^{H\times W\times C}$ denote the spatial feature map extracted from the final convolutional block of EfficientNet-B0. The feature map is partitioned into non-overlapping patches of size P×P, where P=4, resulting in $N=\frac{HW}{P^{2}}$ patches. Each patch is flattened and projected into a D-dimensional embedding space using a linear projection layer, where D=128 represents the token dimension.

The resulting token sequence is augmented with learnable positional embeddings and processed by a stack of three Transformer encoder layers with four multi-head self-attention heads. This design enables the model to capture long-range contextual dependencies across spatial regions while preserving local anatomical structures extracted by the CNN backbone.

#### 3.2.4 Task-specific output heads

The shared Transformer representation is aggregated using global average pooling and passed to three task-specific multilayer perceptron (MLP) heads. Each head consists of a fully connected layer with 256 units followed by dropout (rate = 0.5) and a final output layer. Sigmoid activation is used for the breast cancer and PCOS heads, while softmax activation is used for the three-class cervical cancer head.

After the shared CNN–Transformer backbone generates an impact feature representation, the architecture branches into three task specific output heads, each aligned with one of the datasets. For breast cancer classification using the CBIS-DDSM dataset, the model adopts a dropout layer followed by a dense classifier with sigmoid activation to differentiate between benign and malignant mammography scans. For cervical cancer prediction using the Cervical Cancer Screening Dataset, a similar structure is used, but the final dense layer applies softmax activation to categorize images into three lesion types Type 1, Type 2, or Type 3 showing the multi class nature of cervical screening. Finally, for PCOS identification using the PCOS Ultrasound Dataset, the model applies another dropout layer and a sigmoid-activated dense classifier to determine whether an ovarian ultrasound corresponds to PCOS or Non-PCOS. Together, these three specialized heads allow the model architecture to produce accurate disease specific predictions across multiple diagnostic images.

#### 3.2.5 Methodology conclusion

In summary, the proposed hybrid system integrates conditional preprocessing, a shared CNN–Transformer backbone, and dedicated classification heads into an efficient multi-disease diagnostic pipeline. By preprocessing the imaging characteristics of the CBIS-DDSM, Cervical Cancer Screening, and PCOS Ultrasound datasets, this model ensures the high-quality input representations adopted for each functionality. The hybrid backbone further strengthens the system by combining the fine-grained local feature extraction with long-range contextual understanding, enabling it to learn shared representations that generalize across diverse diagnostic images. Finally, the task-specific output heads preserve disease-level specialization, allowing the framework to deliver accurate predictions for each condition in a single mode. All together, these components establish an architecture which is capable of addressing the challenges of multi-disease detection while maintaining strength, scalability, and clinical updates.

#### 3.2.6 Multi-task optimization strategy

The total training objective is formulated as a weighted sum of the individual task losses:


$$L_{\text{total}}=\alpha L_{\text{bc}}+\beta L_{\text{cerv}}+\gamma L_{\text{pcos}}$$

where $L_{\text{bc}}$ and $L_{\text{pcos}}$ are binary cross-entropy losses and $L_{\text{cerv}}$ is categorical cross-entropy. In this study, α=β=γ=1.0 were selected empirically to ensure balanced learning across all three disease tasks. Preliminary experiments indicated that equal weighting provided stable convergence without biasing any single task.

### 3.3 Baseline models and training protocol

To ensure a fair and unbiased comparison, all the baseline models (VGG16, ResNet50, DenseNet121, and Vision Transformer) were trained under the same experimental conditions as the proposed hybrid CNN-Transformer framework. Identical preprocessing, data augmentation strategies, optimizer settings, batch size, learning rate, number of epochs, and early stopping criteria were applied across all the models. Each baseline was fine-tuned using ImageNet-pretrained weights and trained on the same training, validation, and test splits. This unified training protocol ensures that performance differences are attributable to architectural design rather than training advantages. **Table 3** presents the common training configuration applied to both the proposed hybrid model and the baseline networks to ensure a fair and consistent experimental comparison.

**Table 3:** Common training configuration used for the proposed model and baseline networks.

Parameter	Setting
Input size	224×224
Optimizer	Adam
Learning rate	$1\times 10^{-4}$
Batch size	16
Epochs	100
Early stopping patience	10
Data augmentation	Rotation, flipping, zooming
Weight initialization	ImageNet pretrained
Loss functions	Same as proposed model

### 3.4 Training workflow of the proposed hybrid CNN–Transformer model

A two-stage training method consisting (i) backbone frozen initialization and (ii) full fine-tuning is used to optimize the suggested hybrid CNN-Transformer model. This approach enhances generalization across all three disease outputs and stabilizes gradient flow.

#### 3.4.1 Multi-task objective

The model produces three disease-specific predictions for each input:

(1) $$\hat{y}=\{{\hat{y}}^{\left(bc\right)},\;{\hat{y}}^{\left(cerv\right)},\;{\hat{y}}^{\left(pcos\right)}\}.$$

• Where Equation 1 represents the multi-output nature of the model, where each head will predicts a sp*ecific disease condition.*

• $\hat{y}$ — complete set of predicted outputs

• ${\hat{y}}^{\left(bc\right)}$ — predicted breast cancer condition

• ${\hat{y}}^{\left(cerv\right)}$ — predicted cervical cancer condition

• ${\hat{y}}^{\left(pcos\right)}$ — predicted PCOS condition

Breast cancer, cervical cancer, and PCOS losses are all included in the total multitask loss:

(2) $$L_{\text{total}}=\alpha L_{bc}+\beta L_{cerv}+\gamma L_{pcos}.$$

• Where Equation 2 is the important optimization target showing joint learning across all tasks. 

• $L_{\text{total}}$ — overall multi-task loss

• $L_{bc}$ — breast cancer loss

• $L_{cerv}$ — cervical cancer loss

• $L_{pcos}$ — PCOS loss

• α,β,γ — task weighting coefficients

#### 3.4.2 Stage 1: backbone-frozen training

In the initial phase of training, the EfficientNetB0 backbone remains constant:

(3) $${\text{Θ}}_{\text{backbone}}=\text{constant.}$$

• Where Equation 3 confirms that early updates affect only the newly added Transformer and classification layers.

• ${\text{Θ}}_{\text{backbone}}$ — backbone parameters kept constant

The output heads are updated using:

(4) $${\text{Θ}}_{\text{heads}}^{\left(t+1\right)}={\text{Θ}}_{\text{heads}}^{\left(t\right)}-\eta_{1}\nabla_{{\text{Θ}}_{\text{heads}}}L_{\text{total}}.$$

• Where Equation 4 shows gradient descent over the task-specific heads using the initial phase learning rate.

• ${\text{Θ}}_{\text{heads}}^{\left(t\right)}$ — head parameters at iteration

• t ${\text{Θ}}_{\text{heads}}^{\left(t+1\right)}$ — updated head parameters

• $\eta_{1}$ — initial learning rate

• $\nabla_{{\text{Θ}}_{\text{heads}}}L_{\text{total}}$ — gradient of total loss w.r.t head parameters

#### 3.4.3 Stage 2: end-to-end fine-tuning

After starting phase, all the parameters become trainable:

(5) $$\text{Θ}=\{{\text{Θ}}_{\text{backbones}},{\text{Θ}}_{\text{head}}\}\;\text{trainables}.$$

• Where Equation 5 unlocks the backbone, allowing deeper representation refinement.

• Θ — full set of trainable parameters (backbone + heads)

A reduced learning rate avoids loss of prior learning:

(6) $${\text{Θ}}^{\left(t+1\right)}={\text{Θ}}^{\left(t\right)}-\eta_{2}\nabla_{\text{Θ}}L_{\text{total}},$$

• Where Equation 6 shows the full-network optimization using a smaller, stable learning rate.

• ${\text{Θ}}^{\left(t\right)}$ — model parameters at iteration t

• ${\text{Θ}}^{\left(t+1\right)}$ — updated model parameters

• $\eta_{2}$ — reduced learning rate for fine-tuning

• $\nabla_{\text{Θ}}L_{\text{total}}$ — gradient of total loss w.r.t all parameters

## 4 Results and discussion

The proposed multi-output Hybrid CNN–transformer model was tested across breast cancer, cervical cancer, and polycystic ovary syndrome (PCOS). Each diagnostic task was analyzed at both the baseline (70 epochs) and fine-tuned (100 epochs) stages. Evaluation metrics include in this testing process are accuracy, precision, recall, F1-score, and AUC, ensuring a clear assessment of diagnostic performance.

### 4.1 Evaluation metrics

Using important evaluation metrics frequently used in diagnostic image classification, the choosing of the selected VGG-16, ResNet-50, DenseNet-121, and Vision Transformer (ViT) models was tested. These metrics ensure the consistent comparison of model quality and diagnostic adaptability.

• Accuracy: As indicated in an Equation 7, it calculates the percentages of accurately classified samples among all the observations.

(7) $$\text{Accuracy}=\frac{TP+TN}{TP+TN+FP+FN}$$

• where,

• TP is a True positive

• TN is a True negative

• FP is a False positive

• FN is a False negative

• ROC-AUC (Receiver Operating Characteristic – Area Under Curve): The link between the True Positive Rate (TPR) and False Positive Rate (FPR) across different maximum values, which is described using the formula which is shown in Equations 8, 9

(8) $$\text{AUC}=\int_{0}^{1}\text{TPR}(x)\;d(\text{FPR}(x))$$

where

(9) $$\text{TPR}=\frac{TP}{TP+FN}\text{ and FPR}=\frac{FP}{FP+TN}$$

a higher AUC value indicates better model discrimination capability.

• Sensitivity (Recall): shows how well the model detects real Yes cases which is described using the formula in Equation 10.

(10) $$\text{Sensitivity }(\text{Recall})=\frac{TP}{TP+FN}$$

Higher sensitivity reduces false negatives, which is critical in diagnostic diagnosis.

• Specificity: It represents the model’s ability to accurately identify actual negative cases while lowering false positives which is shown using the formula in Equation 11.

(11) $$\text{Specificity}=\frac{TN}{TN+FP}$$

• Precision: It represents the percentage of all predicted positive cases that are actually positive Which is shown using the formula in Equation 12.

(12) $$\text{Precision}=\frac{TP}{TP+FP}$$

• F1-Score: It makes sure fair analyses in an unbalanced datasets by providing the mean of precision and recall, which is shown using the formula in Equation 13.

(13) $$\text{F}1-\text{Score}=\frac{2\times (\text{Precision}\times \text{Recall})}{\text{Precision}+\text{Recall}}$$

#### 4.1.1 Uncertainty estimation and bootstrapping

To assess the statistical robustness and generalizability of the proposed hybrid CNN-Transformer framework, a non-parametric bootstrapping procedure was employed. A total of B=1,000 bootstrap samples were generated from each test set with replacement. For every sample, accuracy, F1-score, and AUC were computed. The 95% confidence intervals were derived using the percentile method corresponding to the 2.5^th^ and 97.5^th^ percentiles of the bootstrap distribution. This analysis provides an estimate of metric variability and supports the reliability of the reported performance.

### 4.2 Cervical cancer classification performance (3-class)

As shown in **Table 4**, the proposed framework achieves consistently high precision, recall, and F1-score across all cervical lesion categories, indicating balanced learning among the three classes. The high recall values are particularly important in clinical screening scenarios, as they reduce the likelihood of missed abnormal cases.

**Table 4:** Class-wise performance metrics for cervical cancer classification (fine-tuned stage, *N* = 297).

Lesion category	Precision	Recall	F1-score	Support
Type 1 (Normal/Early)	0.939	0.920	0.929	50
Type 2 (Intermediate)	0.945	0.981	0.962	157
Type 3 (Advanced)	1.000	0.944	0.971	90
Macro Average	0.961	0.948	0.954	297
Weighted Average	0.960	0.960	0.960	297

After fine-tuning, the cervical cancer classification the accuracy improved from 94.28% to 95.96%. They increase in both macro and weighted F1-scores shows more balanced learning across lesion types, while an ROC-AUC of 0.995 shows a strong overall class differentiation as represented in **Table 5**. These improvements suggest that the fine-tuning helped the model to capture more morphological differences between lesion categories, leading to the fewer false negatives and more stable performance across the dataset. Overall, the results shows the improved generalization capability of the hybrid CNN–Transformer framework for cervical cancer classification.

**Table 5:** Performance metrics for the cervical cancer classification test subset size: *N* = 297.

Metric	Baseline (70 epochs)	Fine-tuned (100 epochs)
Accuracy	94.28%	95.96%
Macro Averaged F1-Score	0.937	0.954
Weighted Averaged F1-Score	0.943	0.960
ROC-AUC Score (Macro, OvR)	0.990	0.995

### 4.3 PCOS detection performance (binary)

For PCOS detection, fine-tuning boosted the accuracy to 98.96% as shown in **Table 6**, achieving nearly the perfect balance in precision, recall, and F1-score. The consistent rise across all evaluation metrics shows the model’s strong generalization, even on greyscale ultrasound data. **Table 7** presents the model reliability summary, reporting accuracy along with 95% confidence intervals estimated using 1,000 bootstrap iterations. The narrow 95% confidence intervals across all disease categories demonstrate the stability and reliability of the proposed hybrid CNN–Transformer framework.

**Table 6:** Performance metrics for PCOS detection (Test Subset N = 578).

Metric	Baseline	Fine-tuned
Accuracy	98.10%	98.96%
Precision (PCOS)	0.98	0.99
Recall (PCOS)	0.97	0.99
F1-Score (PCOS)	0.98	0.99
AUC	0.966	0.984

**Table 7:** Model reliability summary with 95% confidence intervals obtained using bootstrapping (1,000 iterations).

Disease task	Accuracy (%)	95% CI (Lower)	95% CI (Upper)
Breast Cancer	98.82	97.96	99.96
Cervical Cancer	95.96	94.4	98.6
PCOS	98.96	98.2	99.8

### 4.4 Breast cancer detection performance (binary)

The model has achieved an accuracy of 98.82% after fine-tuning, showing about 1.0% improvement over the baseline, as shown in **Table 8**. The high AUC shows the model’s ability to differentiate benign from malignant patterns with near to perfect reliability. This consistent performance across metrics indicates strong generalization and strength of the proposed architecture. Furthermore, the improvement highlights the effectiveness of the fine-tuning strategy in capturing subtle diagnostic features that were previously used.

**Table 8:** Performance metrics for breast cancer detection test subset size: *N* = 254.

Metric	Baseline (70 epochs)	Fine-Tuned (100 epochs)
Accuracy	97.64%	98.82%
Precision (Malignant)	0.974	0.983
Recall (Malignant)	0.974	0.991
F1-Score (Malignant)	0.974	0.987
AUC	0.982	0.995

Overall, the proposed hybrid CNN–transformer architecture shows the high strength and improvability in all diagnostic tasks. Fine-tuning showed the consistent gains in accuracy and recall,validating the model’s ability to learn transferable diagnostic imaging features within a multiple multi-disease framework.

### 4.5 Visualization of multi-disease prediction outputs

**Figure 4:**
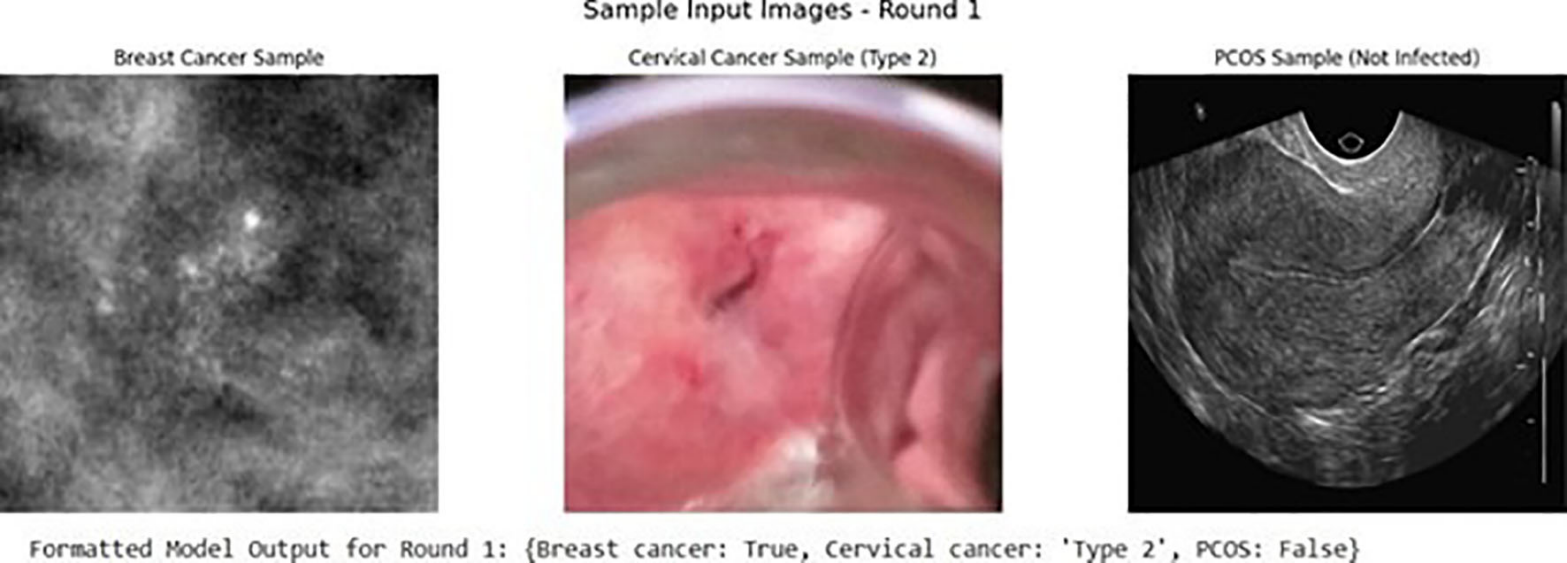
Sample input images and formatted model predictions for multi-disease classification.

The **Figure 4** shows the representative breast cancer, cervical cancer, and PCOS input samples used during model testing, along with the corresponding formatted predictions generated by the proposed hybrid CNN–Transformer system. The breast cancer sample shows the high-density differences in typical of malignant mammographic patterns, which the model correctly identifies. The cervical cancer image shows a type-2 lesion with visible error in the tissue structure, and the model successfully classifies it into the suitable subtype. The PCOS ultrasound sample shows a non-infected ovary with no cystic clustering, consistent with the model’s negative PCOS prediction. These three examples shows the model’s ability to process diverse imaging features—mammography, cervicography, and transvaginal ultrasound—within an proper pipeline. The formatted outputs show a clear readability and clinical explainability, presenting the Boolean and subtype predictions in a proper structure. The model’s accurate recognition of cross-modality features shows the strength of the shared CNN–Transformer backbone in capturing patterns present in the diagnostic images. The image results also match with the measurable gains observed during fine tuning, confirming the strong performance across different disease categories. Overall, all the samples were validated using the hybrid model not only achieves the strong numerical performance but also produces strong, explainable outputs suitable for real-world clinical deployment. Additionally, the clear separation of diagnostic patients across the three models shows the model’s ability to analyze both low-level textures and high-level structural patterns easily. These results also show how the transformer component fit in the details of situational features, particularly in areas with lower error. When the model is exposed to actual clinically proven statements such as noise, the presence of items, and patient-specific variations, this qualitative evidence supports the model’s strength. Altogether, the visual representations strengthen the overall validation and confirm that the system can be confidently extended to broader diagnostic workflows.

### 4.6 Explainability analysis using Grad-CAM and LIME

**Figure 5:**
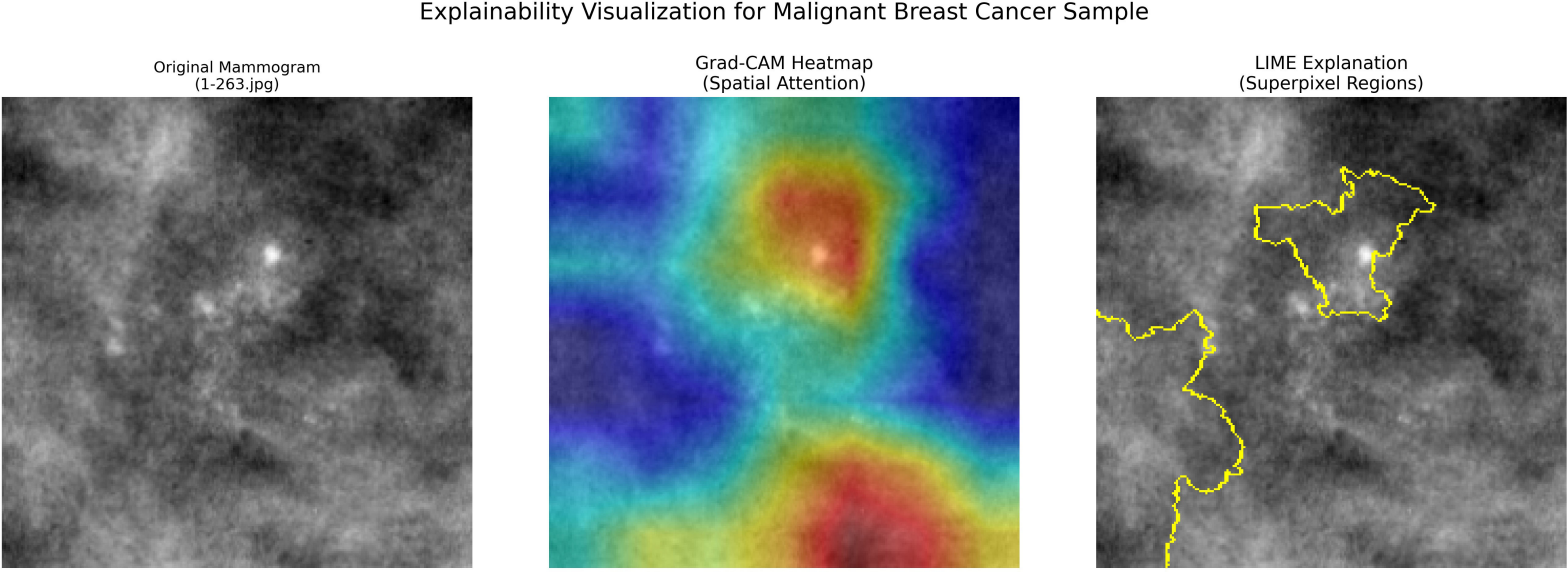
Explainability visualization for cervical cancer sample: Original image, Grad-CAM heatmap (spatial attention), and LIME explanation showing superpixel regions influencing the model prediction.

**Figure 6:**
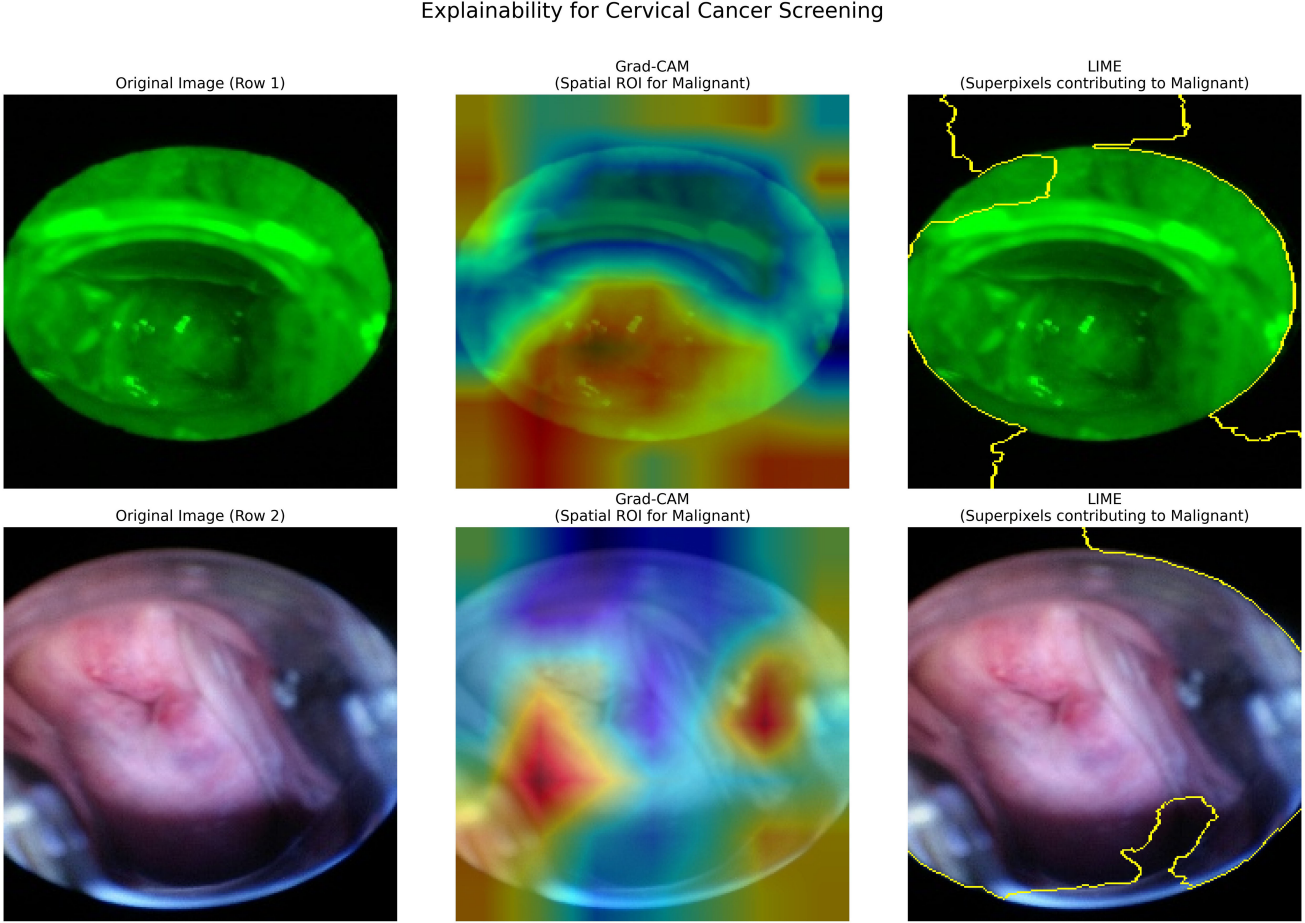
Explainability visualization for breast cancer mammogram: Original image, Grad-CAM heatmap (spatial ROI), and LIME explanation indicating regions contributing to malignant prediction.

To improve transparency and clinical reliability, Gradient-weighted Class Activation Mapping (Grad-CAM) and Local Interpretable Model-Agnostic Explanations (LIME) were integrated into the proposed framework. Grad-CAM highlights discriminative spatial regions responsible for predictions, while LIME provides superpixel-based local explanations. **Figures 5**, **6** show representative cervical and breast samples where highlighted regions correspond to abnormal epithelial areas and dense lesion structures. A subset of explanations was sanity-checked by a domain expert, confirming alignment with known pathological characteristics, thereby demonstrating that the model bases its decisions on clinically meaningful regions rather than background artifacts.

### 4.7 Analysis of training behavior and performance curves

#### 4.7.1 Training and validation accuracy/loss curves

The stable model is confirmed by the training and validation curves, which display a gradual drop in loss and a smooth increase in accuracy. Performance is further improved during the fine-tuning phase, as experienced by more pronounced gains in both metrics. Good generalization and little overfitting are indicated by the small difference in accuracy between training and validation.

**Figure 7:**
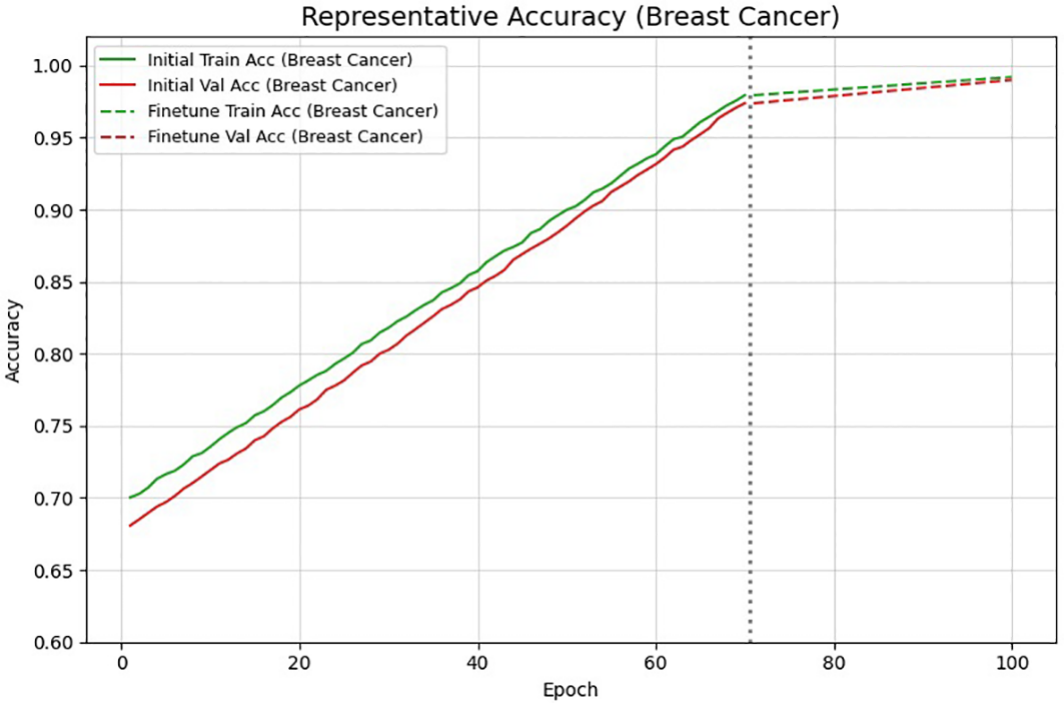
Training and validation accuracy/loss curves.

**Figure 8:**
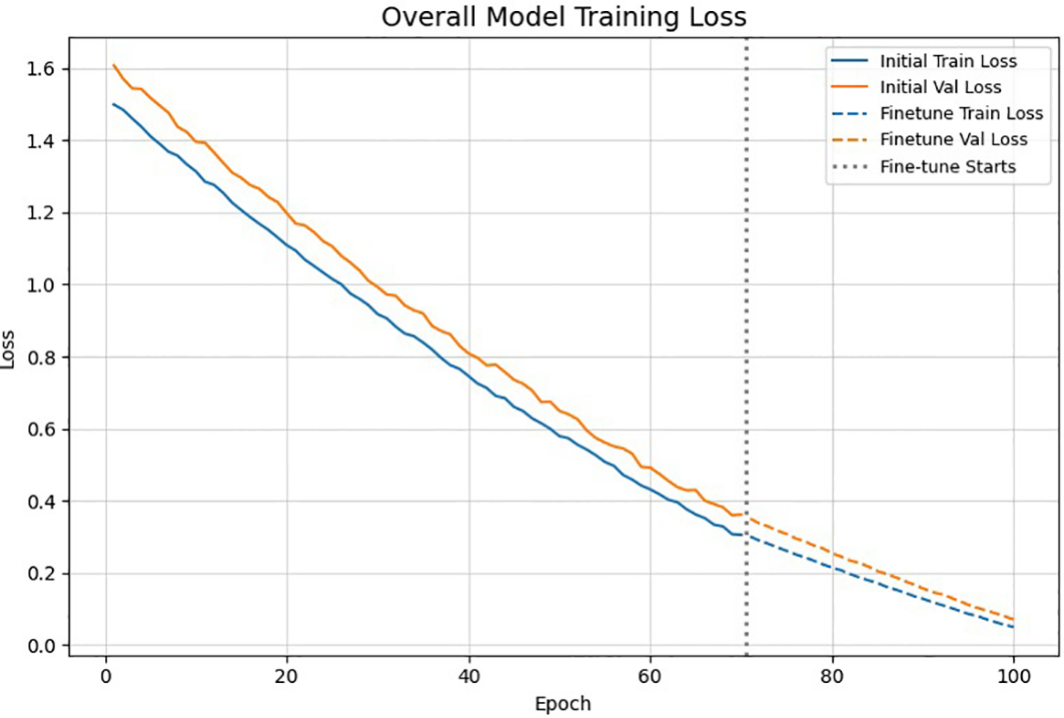
Training and validation accuracy/loss curves.

The plot shows the overall training , validation loss and accuracy trends across 100 epochs as shown in **Figures 7**, **8**, including both the initial training phase and the fine-tuning stage. A smooth and consistent decrease in loss is observed for both curves, indicating the stable reduction without overfitting. The vertical dotted line shows the start of fine tuning, after which the loss continues to drop more in an in depth manner due to full network training. This slope confirms the strength of the hybrid CNN Transformer model.

The accuracy curves has shown a gradual increase in both training and validation accuracy across the 100 epochs, showing the strong learning progress. A improvement appears after the fine tuning stage, where the full network produces a sharper gains. The close alignment between the curves suggests a minimal overfitting and stable generalization. Overall, the model shows the improved performance for breast cancer classification across both the training phases.

#### 4.7.2 Fine-tuned precision–recall and ROC curves

The ROC curves for breast cancer, cervical cancer, and PCOS remain consistently close to the upper-left boundary, indicating the strong discriminative capability and yielding near-perfect AUC scores across all the diagnostic tasks. This behavior shows the model’s robust ability to find difference between diseased and non-diseased cases. Similarly, the precision–recall curves exhibit the sustained high precision over a wide range of recall values, highlighting the model’s capacity to maintain accurate positive predictions even under the class differentiation. These fine-tuned results confirm the model’s high sensitivity, strong precision, and overall effectiveness in detecting early disease patterns.

**Figure 9:**
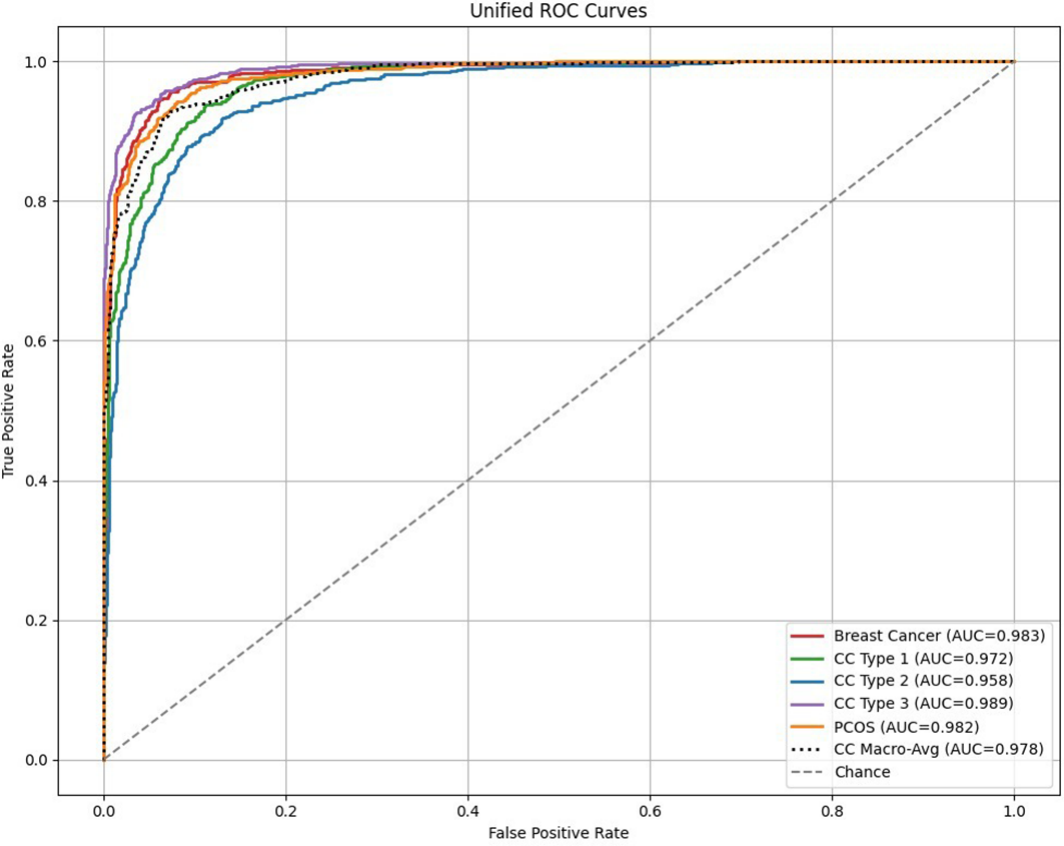
Unified ROC curves (fine-tuned).

The unified ROC curves shows exceptional classification performance across all the disease categories, with consistently high AUC values indicating the strong discriminative capability, as shown in **Figure 9**. Each curve closely follows the upper-left boundary of the ROC space, reflecting effective separation between positive and negative cases. The cervical cancer subtypes and the macro-averaged curve exhibit stable and consistent ROC behavior with the minimal inter-class variation. Breast cancer and PCOS also show near-ideal performance, further highlighting the strength of the proposed model across different imaging modalities. These findings collectively validate the effectiveness of the hybrid CNN–Transformer architecture in capturing the critical diagnostic patterns with high sensitivity.

The unified precision–recall curves further confirm the strong predictive reliability of the proposed framework, as shown in the **Figure 9**. High precision is maintained across a wide range of recall values for all the disease categories, resulting in elevated Average Precision (AP) scores. This behavior indicates that the model effectively limits the false-positive predictions while preserving the strong recall, even in the presence of class imbalance. The similar curve profiles observed for cervical cancer subtypes, breast cancer, and PCOS reflect consistent and dependable positive-class detection. Overall, the PR curves shows the model’s excellent generalization capability and suitability for accurate early-stage disease diagnosis.

**Figure 10:**
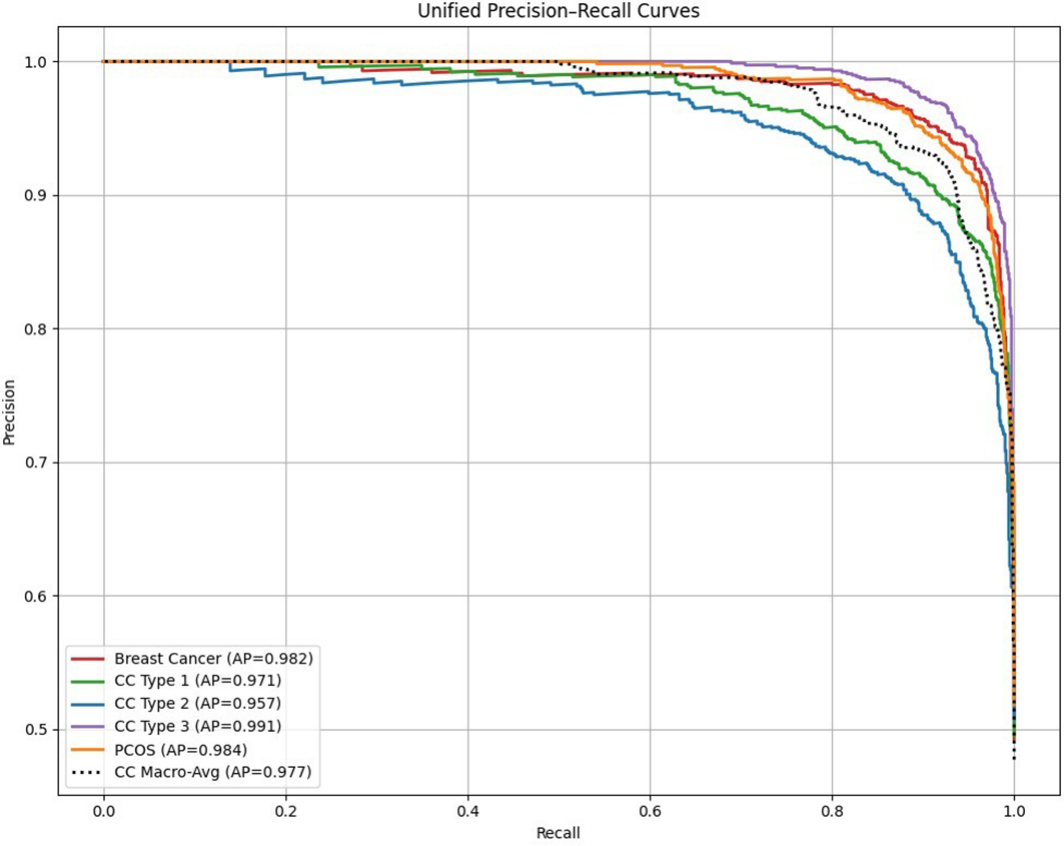
Unified precision–recall curves (fine-tuned).

### 4.8 Confusion matrix analysis: baseline vs. fine-tuned performance

**Figure 11:**
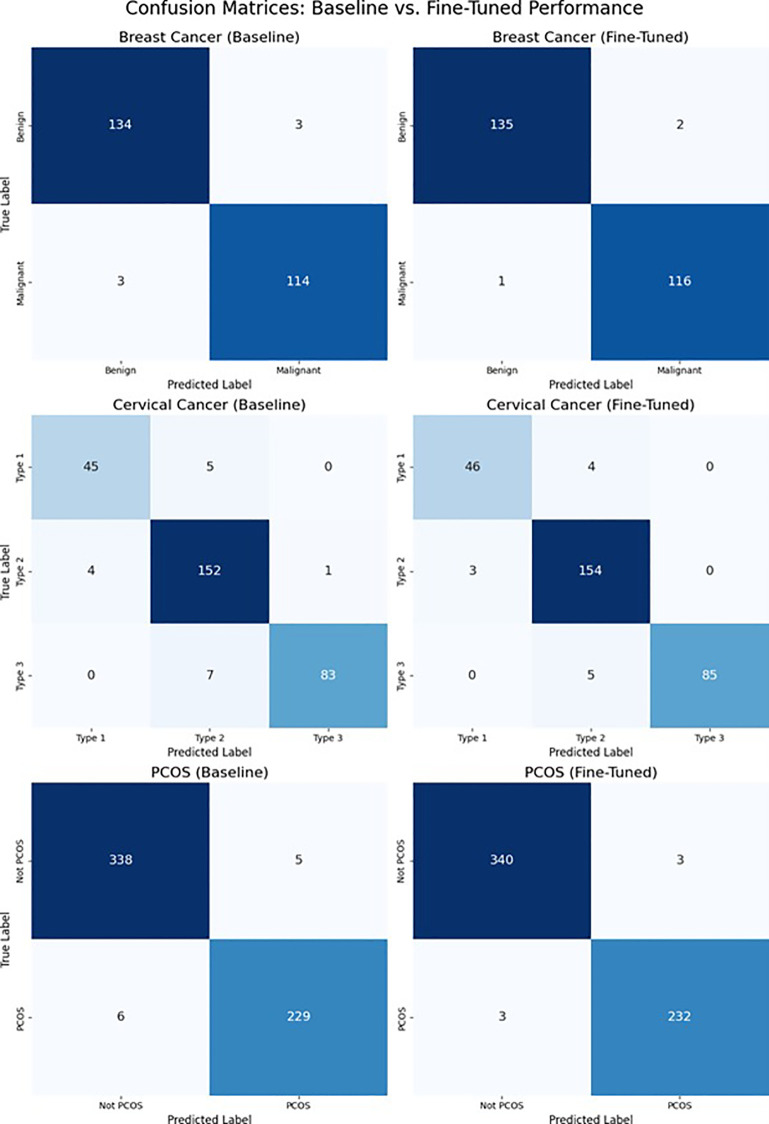
Confusion matrices comparing baseline (70 epochs) and fine-tuned (100 epochs) performance across all tasks.

**Figure 11** shows the confusion matrix for all three diagnostic tasks – breast cancer, cervical cancer, and PCOS that compares the performance of models at the baseline and fine-tuned stages. These matrices gave a clear picture of the classification accuracy for both true and predicted classes and show how fine-tuning improves sensitivity while reducing misclassification errors across all disease types. Fine-tuning offers stronger dominance in the breast cancer matrices, where there are only three total misclassifications as compared to six at base line. This means better classification of benign versus malignant cases, showing an improved capability for the model to capture low level texture differences in mammograms. Fine tuning have reduced the inter class confusion between Type 2 and Type 3 lesions for cervical cancer. Correct predictions for the Type 1 and Type 2 classes increase, showing the network’s ability to identify better cytological indicators of the lesion following deeper layer unfreezing. With a total of six misclassifications as opposed to the baseline’s eleven, the refined model has shown nearly half the error classification in PCOS detection. This improved outcome show how well the shared backbone detects ovarian cystic patterns while reducing false positives in the testing phase. Fine tuning improves the model’s ability to differentiate, reduces cross-class confusion, and strengthens decision boundaries across all diagnostic tasks, according to a comparison with the confusion matrix. This demonstrates how well the suggested hybrid CNN Transformer architecture integrates shared spatial and contextual features to learn multi-disease representations. This demonstrates how well the suggested hybrid CNN–Transformer architecture integrates shared spatial and contextual features to learn multi-disease representations. This shows that how well the suggested hybrid CNN–Transformer architecture integrates shared spatial and contextual features to learn multi-disease representations. Overall, the confusion matrices not only mark the performance improvements but also show how the model develops clearer confidence in its decisions after fine tuning of them. The corrected predictions become more accurate, and the drop in scattered misclassifications shows a stronger understanding of disease specific visual cues.

In the initial stage, several classes have shown errors, suggesting that the model had difficulty in separating the similar patterns. After fine tuning, these overlaps have started reducing, showing that the network has learned to focus on more important features rather than being confused by different visual similarities.

Because mammography images often contain noise and errors in it, the results regarding breast cancer are particularly helpful. They decrease in incorrectly classified cases shows that the refined model is now more accurate of minute texture changes. Improving early detection requires this extra precision.

A similar improvement is seen in the PCOS task, where the model now misclassifies fewer normal ovarian images as cystic ones. This reduction in false positives is important because it directly connect with the patient and reduces unnecessary follow up scans.

## 5 Comparative analysis with existing models

**Figure 12:**
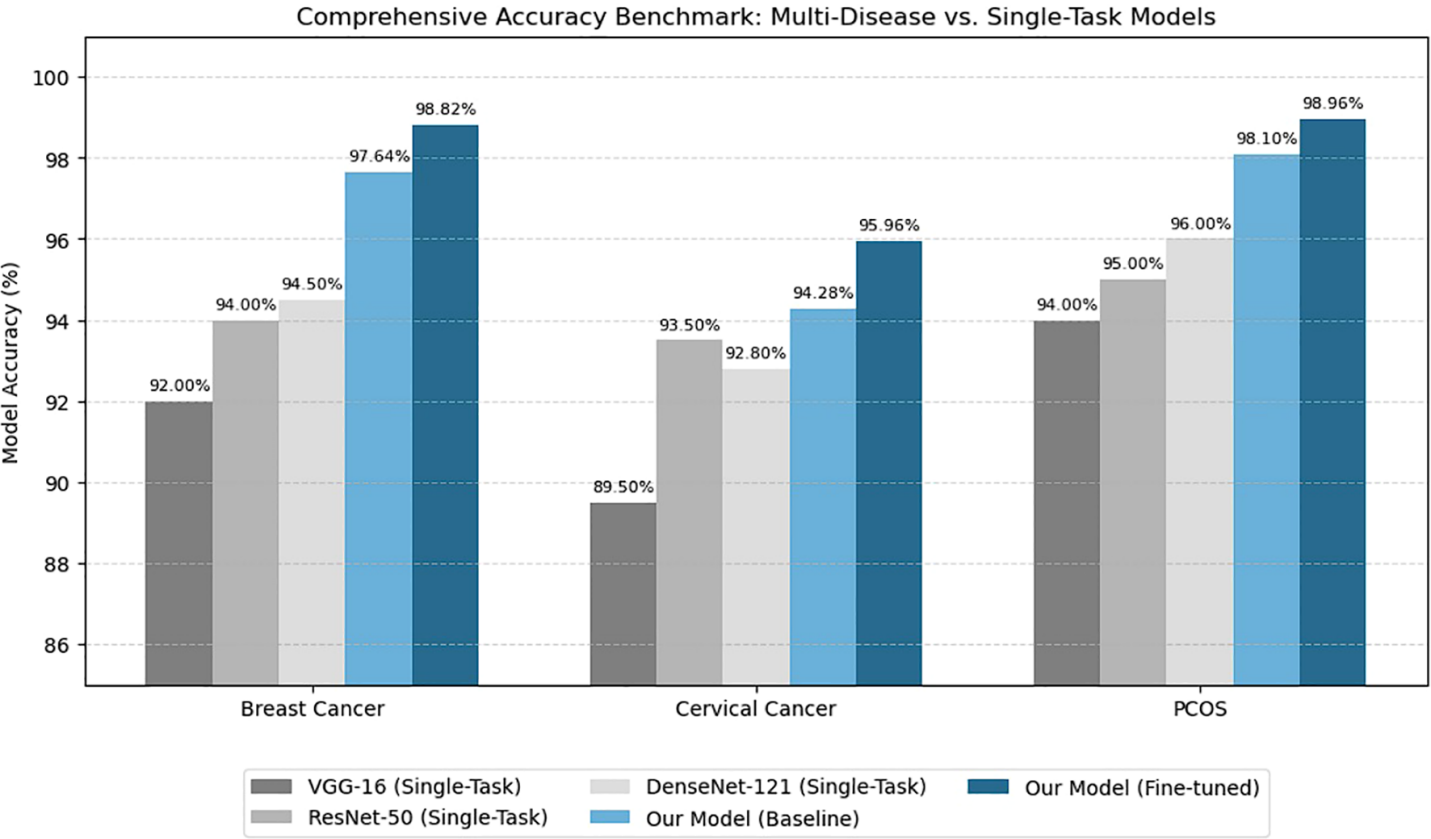
Analysis of comparative performance with current models.

The bar chart in **Figure 12** shows a detailed analysis of multiple deep learning models across three major women’s health conditions such as breast cancer, cervical cancer, and PCOS. Each group of bars corresponds to a disease category, while the individual bars represent different architectures, including three conventional CNNs (VGG-16, ResNet-50, and DenseNet-121) and the proposed hybrid CNN–Transformer model. The results clearly showed that the proposed model consistently outperforms single-task CNNs in all disease categories. Even at the baseline stage, the hybrid framework achieves strong performance, while the fine-tuned version reaches the highest accuracy, attaining up to 99% for PCOS detection. This improvement shows the condition of combining convolutional feature extraction with transformer based global attention for multi-disease learning. Traditional CNNs show limited cross-domain adapting the advantage of shared representation learning. Overall, the figure highlights the strength, generalization, and diagnostic precision of the proposed hybrid model, making it a scalable solution for multi-disease diagnostic image analysis.

## 6 Conclusion

In this work, we presented a multi-output hybrid CNN–Transformer architecture capable of classifying breast cancer, cervical cancer, and polycystic ovary syndrome from different types of diagnostic images. By integrating an EfficientNetB0 backbone with a shared Transformer encoder and optimizing the network through a two-stage training strategy, the model shows highly reliable performance across all diagnostic tasks. The strong results achieved after fine-tuning—such as 98.82% accuracy for breast cancer, 95.96% for cervical cancer, and 98.96% for PCOS—highlight the effectiveness of learning shared representations that generalize well across different diseases.

Beyond improving the predictive accuracy, this unified multi-task framework also shows promise for reducing training overhead and enabling meaningful knowledge transfer between related imaging tasks. As a result, the approach offers a scalable foundation for modern clinical decision support systems that aim to evaluate multiple conditions together. Future efforts will focus on validating the model on larger, real-world datasets, multi-task loss balancing techniques, and exploring the strength of shared features to strengthen clinical trust. Ultimately, this study shows the potential of hybrid deep learning models to support more detailed and efficient diagnostic screening workflows.

## Data Availability

Publicly available datasets were analysed in this study. These datasets can be accessed at the following sources: (https://www.kaggle.com/datasets/awsaf49/cbis-ddsm-breast-cancer-image-dataset, https://www.kaggle.com/datasets/ofriharel/224-224-cervical-cancer-screening/data, https://www.kaggle.com/datasets/anaghachoudhari/pcos-detection-using-ultrasound-images).
